# The Development of the Pediatric NAFLD Fibrosis Score (PNFS) to Predict the Presence of Advanced Fibrosis in Children with Nonalcoholic Fatty Liver Disease

**DOI:** 10.1371/journal.pone.0104558

**Published:** 2014-08-14

**Authors:** Naim Alkhouri, Sana Mansoor, Paola Giammaria, Daniela Liccardo, Rocio Lopez, Valerio Nobili

**Affiliations:** 1 Department of Pediatric Gastroenterology and Hepatology, Cleveland Clinic, Cleveland, Ohio, United States of America; 2 Digestive Disease Institute, Cleveland Clinic, Cleveland, Ohio, United States of America; 3 Department of Quantitative Health Sciences, Cleveland Clinic, Cleveland, Ohio, United States of America; 4 The Liver Unit, Bambino Gesu Children’s Hospital, Rome, Italy; BIDMC, United States of America

## Abstract

**Background:**

Noninvasive hepatic fibrosis scores that predict the presence of advanced fibrosis have been developed and validated in adult patients with NAFLD. The aims of our study were to assess the utility of commonly used adult fibrosis scores in pediatric NAFLD and to develop a pediatric specific fibrosis score that can predict advanced fibrosis.

**Methods:**

Consecutive children with biopsy-proven NAFLD were included. Fibrosis was determined by an experienced pathologist (F0–4). Advanced fibrosis was defined as fibrosis stage ≥3. The following adult fibrosis scores were calculated for each child: AST/ALT ratio, AST/platelet ratio index (APRI), NAFLD fibrosis score (NFS), and FIB-4 Index. Multivariable logistic regression analysis was performed to build a new pediatric model for predicting advanced fibrosis.

**Results:**

Our cohort consisted of 242 children with a mean age of 12.4±3.1 years and 63% were female. 36 (15%) subjects had advanced fibrosis. APRI and FIB-4 were higher in patients with advanced fibrosis compared to those with fibrosis stage 0–2; however, AST/ALT ratio and NFS were not different between the two groups. We used our data to develop a new model to predict advanced fibrosis which included: ALT, alkaline phosphatase, platelet counts and GGT. The multivariable logistic regression model (z) was defined as follows: z = 1.1+(0.34*sqrt(ALT))+(0.002*alkaline phosphatase) – (1.1*log(platelets) – (0.02*GGT). This value was then converted into a probability distribution (p) with a value between 0 to 100 by the following formula: p = 100×exp(z)/[1+exp(z)]. The AUCROC for this model was 0.74 (95% CI: 0.66, 0.82). This was found to be significantly better than APRI, NAFLD Fibrosis Score and FIB-4 Index.

**Conclusion:**

Noninvasive hepatic fibrosis scores developed in adults had poor performance in diagnosing advanced fibrosis in children with NAFLD. We developed a new pediatric NAFLD fibrosis score with improved performance characteristics.

## Introduction

Due to the epidemic of childhood obesity, nonalcoholic fatty liver disease (NAFLD) has become the most common chronic liver disease in children [1], [2]. Pediatric NAFLD is relatively benign but some children can develop major hepatic complications including cirrhosis and the need for liver transplantation [3]. The presence of advanced fibrosis, which includes bridging fibrosis and cirrhosis, may be the most important factor in determining the prognosis of NAFLD and its risk of progression to end-stage liver disease. Liver biopsy remains the gold standard for assessing NAFLD severity and staging of fibrosis [4]; however, with a disease that affects approximately 10% of American children, performing a liver biopsy is neither practical nor ethical. Moreover, children with bridging fibrosis are very likely to progress to developing cirrhosis in the absence of effective treatment for NAFLD which requires close monitoring for signs of portal hypertension and hepatic decompensation. Therefore, there is an urgent need to develop simple noninvasive tests to identify children with advanced fibrosis. Several noninvasive fibrosis scoring systems comprised of routinely measured clinical and laboratory variables have been developed in adult patients with NAFLD to identify those with advanced fibrosis including the AST/ALT ratio, NAFLD fibrosis score (NFS), the AST/platelet ratio index (APR), and the FIB4-score [5]. However, recent data from our group and others have suggested that these adult scores may not be accurate to predict advanced fibrosis in children [6], [7].

The aims of the current study were to assess the utility of commonly used adult fibrosis scores in a large cohort of pediatric patients with biopsy-proven NAFLD and to develop a pediatric specific fibrosis score that can predict advanced fibrosis.

## Materials and Methods

### Patients

A written informed consent was obtained from the parents, caretakers, or guardians on behalf of the minors/children enrolled in the study. Consecutive patients diagnosed with NAFLD seen at the Bambino Gesù Children’s Hospital were included in the study. Study was approved by Ethics committee of the Bambino Gesù Children’s Hospital and Research Institute in Rome, Italy. Inclusion criteria were persistently elevated serum aminotransferase levels, diffusely hyperechogenic liver on ultrasonography suggestive of fatty liver, and biopsy consistent with the diagnosis of NAFLD [8], [9]. Exclusion criteria were hepatic virus infections (hepatitis A, B, C, and D, cytomegalovirus and Epstein-Barr virus), alcohol consumption, history of parenteral nutrition, and use of drugs known to induce steatosis (e.g. valproate, amiodarone or prednisone) or to affect body weight and carbohydrate metabolism. Autoimmune liver disease, metabolic liver disease, Wilson disease, celiac disease and α-1-antitrypsin deficiency were ruled out using standard clinical, laboratory and histological criteria. A blood sample for laboratory tests was taken within one week of the liver biopsy.

The body mass index (BMI) and its standard deviation score (Z-score) were calculated [10], [11]. The metabolic syndrome (MetS) was defined as the presence of ≥3 of the following 5 criteria [12]: abdominal obesity as defined by a waist circumference ≥90^th^ percentile for age [13]; hypertriglyceridemia as defined by TG >95^th^ percentile for age and sex [14]; low HDL cholesterol as defined by <5^th^ percentile for age and sex [14]; elevated blood pressure (BP) as defined by systolic or diastolic BP>95^th^ percentile for age and sex [15]; and impaired fasting glucose, impaired glucose tolerance (IGT) or known type 2 diabetes mellitus as described in detail elsewhere [16]. The homeostasis model assessment index of insulin resistance (HOMA-IR) was calculated as surrogate markers of insulin sensitivity [17].

### Liver Histology and Noninvasive Fibrosis Scores

The clinical indication for biopsy was either to assess the presence of NASH and degree of fibrosis or other likely independent or competing liver diseases. Liver biopsy was performed in all children, after an overnight fast, using an automatic core biopsy 18 Gauge needle (Biopince, Amedic, Sweden) under general anaesthesia and ultrasound guidance. A Sonoline Omnia Ultrasound machine (Siemens, Germany) equipped with a 5-MHz probe (5.0 C 50, Siemens) and a biopsy adaptor was employed. Two biopsy passes within different liver segments were performed for each subject. The length of liver specimen (in millimetres) was recorded. Only samples with a length ≥15 mm and including at least 6 complete portal tracts [18] were considered adequate for the purpose of the study. Biopsies were evaluated by a single pathologist who was blinded to clinical and laboratory data. Biopsies were routinely processed (*i.e.*, formalin-fixed and paraffin-embedded) and sections of liver tissue, 5 µm thick, were stained with Hematoxylin-Eosin, Van Gieson, PAS-D, and Prussian blue stain. Liver biopsy features were graded according to the NAFLD activity scoring (NAS) system proposed by Kleiner et al [19]. Fibrosis was scored as 0 =  none; 1 =  periportal or perisinusoidal fibrosis; 2 =  perisinusoidal and portal/periportal fibrosis; 3 =  bridging fibrosis; and 4 =  cirrhosis. Advanced fibrosis was defined as fibrosis stage ≥3.

APRI was calculated as AST/ULN (upper limit of normal)/Platelets×100 [20]. The AST/ALT ratio was calculated as ratio of AST to ALT values. The FIB –4 index was calculated using the following formula: FIB - 4 =  (Age×AST)/[Platelet count (10.9/L)×√ALT] [21] [18]. NAFLD Fibrosis Score (NFS) was obtained using the formula: −1.675+0.037×age (years)+0.094×BMI (kg/m2)+1.13×IFG/diabetes (yes = 1, no = 0)+0.99×AST/ALT ratio –0.013×platelet (×109/l) –0.66×albumin (g/dl) [22].

### Statistical analysis

Descriptive statistics were computed for all variables. These include means, standard deviations and percentiles for continuous variables and frequencies and percentages for categorical factors. A univariable analysis was done to assess differences between subjects with and without advanced fibrosis; ANOVA or the non-parametric Kruskal-Wallis tests were used to compare continuous and ordinal factors and Pearson’s chi-square tests were used to compare categorical variables. Multivariable logistic regression analysis was performed to build a model for prediction of advanced fibrosis. An automated stepwise variable selection method performed on 1000 bootstrap samples was used to choose the final model. All non-invasive factors were assessed and the 4 variables with highest inclusion rates were included in the final model.

Discrimination was used for internal model validation; this measures the ability to rank patients by risk of advanced fibrosis such that patients with a higher predicted risk are more likely to have advanced fibrosis. Discrimination was measured by the area under the Receiver Operating Characteristics curve (AUROC). The method described by Harrell [23] was used to compute the validation metric with over-fitting bias correction through bootstrap resampling. A thousand bootstrap samples (B = 1000) were drawn from the original data set and a new model with the same model settings was built on each bootstrap resample. Prediction on patients that were not chosen in the resample was calculated. An optimism factor was calculated over the 1000 new models and the bias-corrected validation metric was obtained by subtracting this optimism value from the AUROC directly measured from the original model. A *P*<0.05 was considered statistically significant. All analyses were performed using SAS (version 9.2, The SAS Institute, Cary, NC) and R (version 3.0.1, The R Foundation for Statistical Computing, Vienna, Austria; packages used: Hmisc and ROCR).

## Results

### Patient Characteristics

A total of 242 pediatric patients with biopsy-proven NAFLD were included in the analysis. 63% were female and the mean age was 12.4±3.1 years. The majority were obese (85%) with a mean BMI percentile of 93.4±7.6. MetS and its individual components were commonly present in our cohort (MetS in 53%). Thirty six patients (15%) had advanced fibrosis F ≥3 (only two patients with cirrhosis). None of the patient had complications from cirrhosis or portal hypertension. Table 1 presents a summary of patient clinical and laboratory characteristics. Hypertension, higher triglycerides, ALT, and AST were significantly associated with presence of advanced fibrosis (p value <0.005 for all).

**Table 1:** Demographic and Clinical Characteristics.

Factor	Overall(N = 242)	Fibrosis stage 0–2(N = 206)	Fibrosis stage 3–4(N = 36)	p-value
**Male (%)**	90(37.2)	79(38.3)	11(30.6)	0.37
**Age at first visit (years)**	12.4±3.1	12.3±3.1	13.1±2.6	0.12
**BMI Percentile**	93.4±7.6	93.4±7.5	93.3±8.4	0.91
**Obese (%)**	205(84.7)	174(84.5)	31(86.1)	0.80
**Low HDL (%)**	127(52.5)	108(52.4)	19(52.8)	0.97
**Hypertriglyceridemia (%)**	154(63.6)	130(63.1)	24(66.7)	0.68
**Hypertension (%)**	77(31.8)	57(27.7)	20(55.6)	**<0.001**
**IGT/Diabetes (%)**	95(39.3)	76(36.9)	19(52.8)	0.072
**Metabolic syndrome (%)**	120(53.1)	107(51.9)	13(65.0)	0.26
**Cholesterol (mg/dL)**	156.7±29.9	156.0±29.8	160.7±30.6	0.39
**HDL (mg/dL)**	54[40,73]	55.5[40,73]	52.5[43,72]	0.86
**Triglycerides (mg/dL)**	90[66,122]	88.5[63,118]	118.0[77,136.5]	**0.006**
**ALT (IU/L)**	75[55,99]	72[52,89]	100.5[72.5,122.5]	**<0.001**
**AST (IU/L)**	49[40,66]	48[39,65]	57.5[48,87.5]	**0.002**
**GGT (IU/L)**	25[19,38]	25[19.0,37]	27[20.5,45.5]	0.19
**Total bilirubin (mg/dL)**	0.66±0.24	0.67±0.24	0.64±0.22	0.58
**Albumin (g/dL)**	4.6±0.54	4.6±0.57	4.6±0.36	0.79
**Alkaline phosphatase (IU/L)**	629.5[481,790]	626[481,771]	714[471.5,857.5]	0.28
**Platelet count (10^9^/L)**	295.5[261,343]	296.5[263,348]	289.5[241,332]	0.16
**HOMA-IR**	2.2[1.4,3.2]	2.1[1.4,3.1]	2.6[1.7,3.5]	0.051

Values presented as Mean ± SD, Median [P25, P75], or N (column %).

P-values: a = ANOVA, b = Kruskal-Wallis test, c = Pearson’s chi-square test. P-values in bold are statistically significant.

### Histological Features of NAFLD

Table 2 presents a summary of the histological features of the biopsy specimens. Approximately 90% of patients had some degree of lobular inflammation while two-thirds revealed at least mild portal inflammation. Ballooning was present in only 50% of patients. Subjects with advanced fibrosis were more likely to have higher grades of steatosis, lobular inflammation, portal inflammation, and ballooning, p value <0.05 for all. The mean NAS was 3.9+1.8 (significantly higher in patients with advanced fibrosis compared to fibrosis stage 0–2, 6±1.5 and 3.5±1.5, respectively; p value <0.001).

**Table 2:** Histological Features and NAFLD Activity Score.

Factor	Overall(N = 242)	Fibrosis stage 0–2(N = 206)	Fibrosis stage 3–4(N = 36)	p-value
**Steatosis**				***<0.001***
**. <5%**	2(0.83)	0(0.0)	2(5.6)	
**. 5–33%**	85(35.1)	85(41.3)	0(0.0)	
**. 34–65%**	80(33.1)	69(33.5)	11(30.6)	
**. > = 66%**	75(31.0)	52(25.2)	23(63.9)	
**Lobular inflammation**				***<0.001***
**. None**	20(8.3)	20(9.7)	0(0.0)	
**. <2 under 20x**	166(68.6)	159(77.2)	7(19.4)	
**. 2–4 under 20x**	54(22.3)	25(12.1)	29(80.6)	
**. >4 under 20x**	2(0.83)	2(0.97)	0(0.0)	
**Portal inflammation**				***0.030***
**. None**	90(37.2)	81(39.3)	9(25.0)	
**. Mild**	134(55.4)	113(54.9)	21(58.3)	
**. More than mild**	18(7.4)	12(5.8)	6(16.7)	
**Ballooning**				***<0.001***
**. None**	121(50.4)	118(57.8)	3(8.3)	
**. Few**	46(19.2)	39(19.1)	7(19.4)	
**. Many**	73(30.4)	47(23.0)	26(72.2)	
**NAS**	3.9±1.8	3.5±1.5	6.0±1.5	***<0.001***

Values presented as Mean ± SD, or N (column %).

P-values in bold are statistically significant.

### Adult Noninvasive Fibrosis Scores in Children with NAFLD

Of the commonly used fibrosis scores shown in Table 3, APRI and FIB-4 index were found to be significantly higher in patients with advanced fibrosis (APRI values were 0.51 [0.39, 0.80] in children with advanced fibrosis and 0.40 [0.30, 0.55] in those without, p value = 0.001. FIB-4 index values were 0.26±0.15 and 0.32±0.14, respectively, p value = 0.037). Of note, these values for both APRI and FIB-4 index in children with advanced fibrosis were much lower than the proposed cutoff values to identify advanced fibrosis in adult patients with NAFLD (1.5 for APRI and 2.67 for FIB-4 index). More importantly, there was no difference in the AST/ALT ratio or the NFS between those with and without advanced fibrosis.

**Table 3:** Adult Noninvasive Fibrosis Scores in Children with NAFLD.

Score	Overall(N = 242)	Fibrosis stage 0–2(N = 206)	Fibrosis stage 3–4(N = 36)	p-value
**AST/ALT ratio**	0.65[0.52,0.87]	0.66[0.54,0.88]	0.55[0.47,0.79]	0.059
**APRI**	0.41[0.30,0.56]	0.40[0.30,0.55]	0.51[0.39,0.80]	**0.001**
**NAFLD Fibrosis Score**	−4.6±1.3	−4.7±1.4	−4.3±1.2	0.14
**FIB-4 Index**	0.27±0.15	0.26±0.15	0.32±0.14	**0.037**
**PNFS**	14.9±12.5	13.2±10.1	24.7±18.7	**<0.001**

Values presented as Mean ± SD or Median [P25, P75].

P-values in bold are statistically significant.

### The Development of the Pediatric NAFLD Fibrosis Score (PNFS)

**Figure 1:**
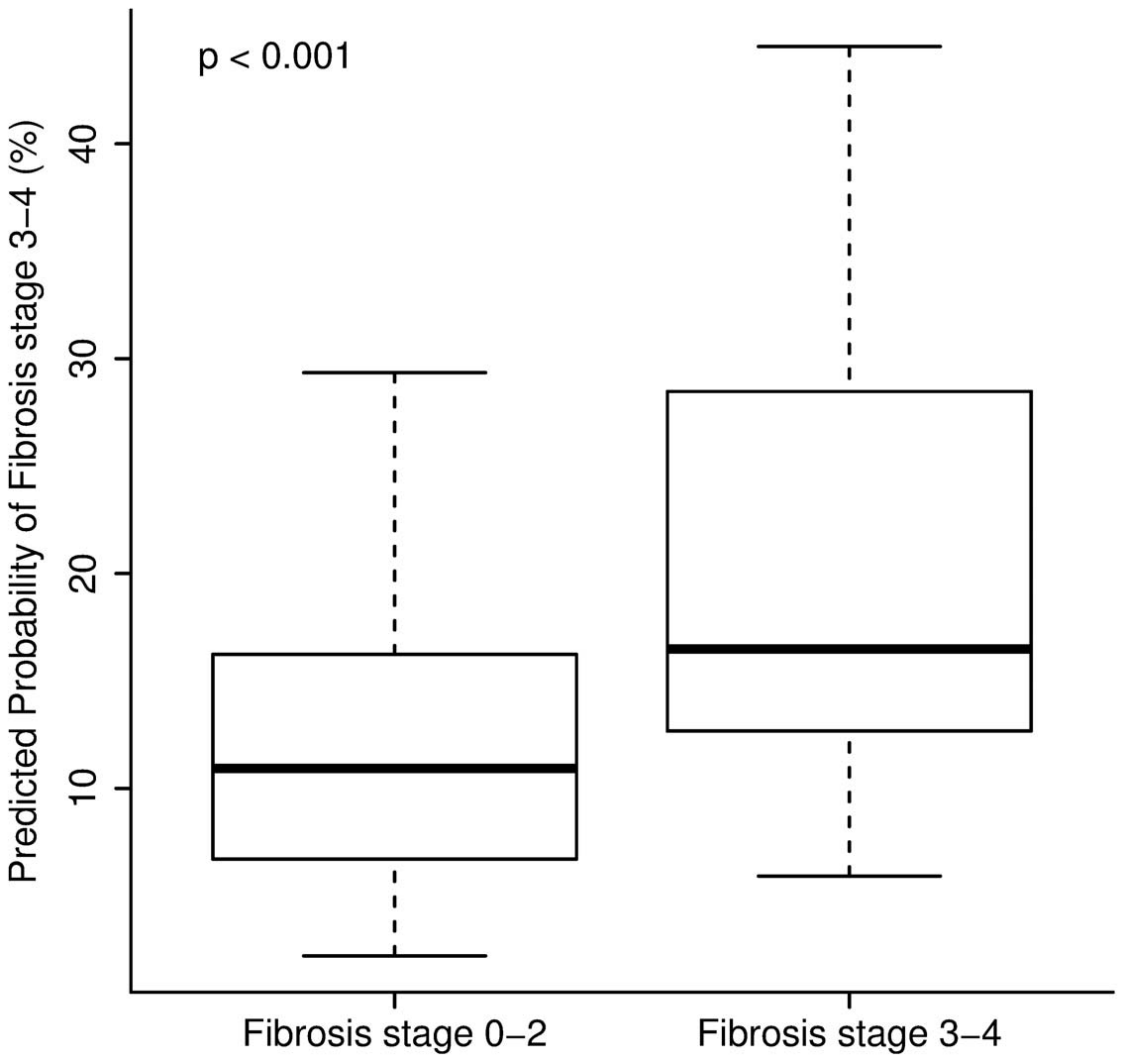
Pediatric NAFLD Fibrosis Score Values are significantly higher in children with advanced fibrosis. The lower boundary of the box-and-whisker plot corresponds to the 25th percentile, the line within the box to the median, and the upper boundary of the box to the 75th percentile. The whiskers extend to the most extreme data point which is no more than 1.5 times the interquartile range from the box.

We performed a multivariable logistic regression analysis to build a model for prediction of advanced fibrosis using available clinical variables. The final proposed model consists of ALT, alkaline phosphatase, platelet count and GGT. The multivariable logistic regression model (z) for prediction of advanced fibrosis was defined as follows:
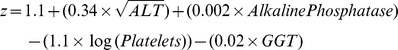



This value was then converted into a probability distribution (p) with a value between 0 to 100 by the following formula:
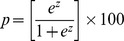



The mean PNFS for patients with stage 0–2 fibrosis was 13.2±10.1 compared to 24.7±18.7 for those with advanced fibrosis, p value <0.001, as shown in Figure 1.

**Figure 2:**
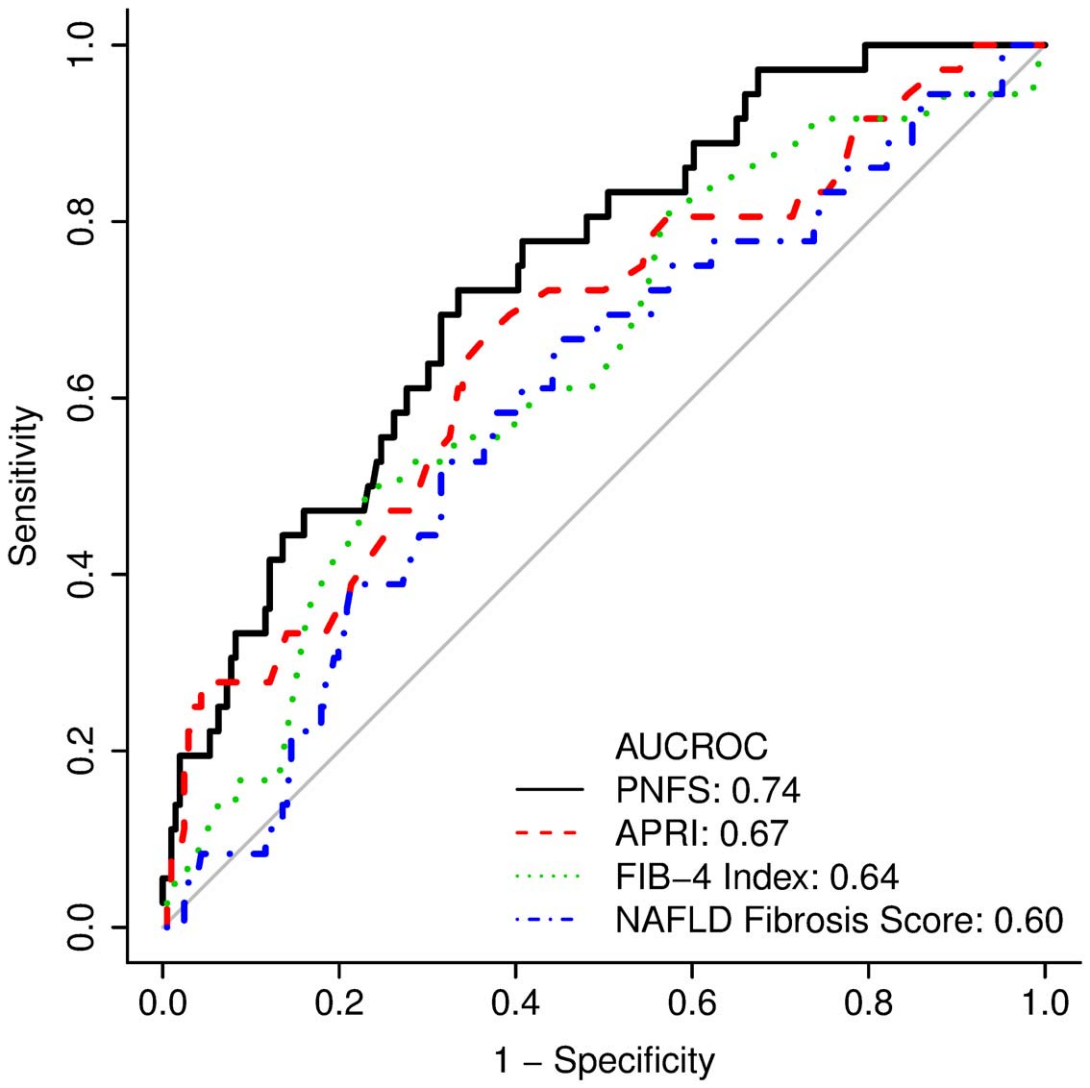
Performance characteristics of the Pediatric NAFLD Fibrosis Score (PNFS) in comparison to APRI, FIB-4, and NFS. The new model (PNFS) was more accurate for predicting advanced fibrosis than APRI, FIB-4 index and NAFLD fibrosis score as assessed by the area under the ROC curve (AUROC).

The AUROC for the new PNFS model was 0.74 (95% CI: 0.66, 0.82) which was higher than the AUROC for APRI, NAFLD Fibrosis Score and FIB-4 Index (Figure 2). A cutoff of 26% would provide specificity, sensitivity, positive and negative predictive values of 92%, 31%, 41% and 88%, respectively, for predicting advanced fibrosis. The proportion of false positives would be 8% for the 26% cut off value (high specificity). On the other hand, a cutoff of 8% would provide specificity, sensitivity, positive and negative predictive values of 33%, 97%, 20% and 99%, respectively. The proportion of false negatives would be 3% for the 8% cutoff value (high sensitivity).

**Figure 3:**
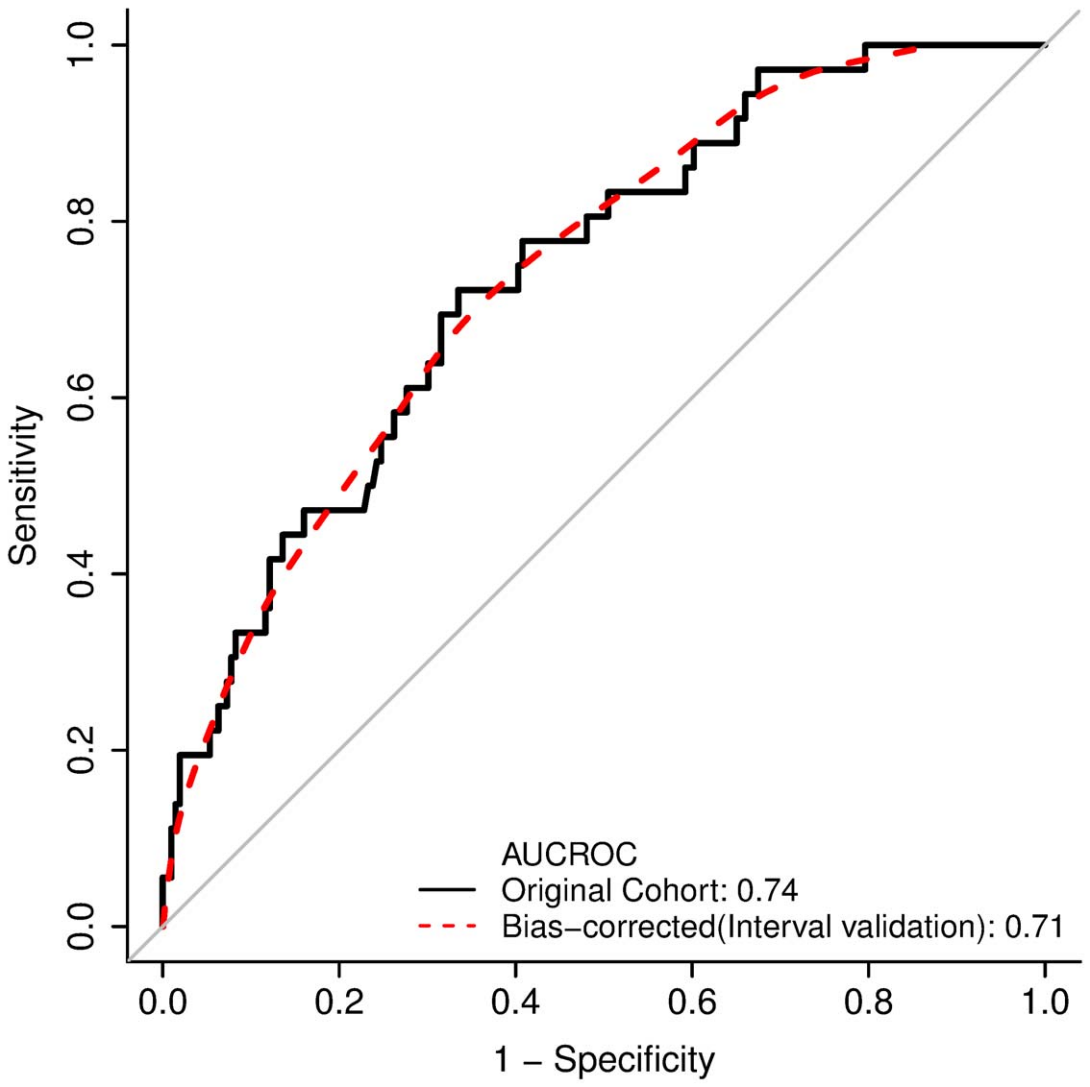
Bootstrap Validation of the Proposed PNFS Model. Internal validation of the model using bootstrapping showed an area under the ROC curve (AUROC) of 0.71.

Internal validation of the model using bootstrapping showed an AUROC of 0.71 (Figure 3).

An online calculator was developed to allow for increased ease of utilizing PNFS in pediatric patients with NAFLD. This calculator can be found at:

http://www.r-calc.com/calculator.aspx?calculator_id=JYAVKOWT.

In Table 4, two study patient examples (with and without biopsy proven advanced fibrosis) are provided to reflect the implementation of PNFS to assess for the probability of having advanced fibrosis.

**Table 4:** Utilization of the PNFS in Two Patients with and without Advanced Fibrosis.

	ALT	Alkaline Phosphatase	Platelet Count	GGT	Probability	Fibrosis
**Patient One**	280	845	180	50	77.1	Fibrosis 3–4
**Patient Two**	28	652	343	15	5.0	Fibrosis 0–2

## Discussion

As the prevalence of pediatric NAFLD continues to remain high especially among obese children, the development of reliable and simple scoring systems that can predict the presence of advanced disease is crucial. The main findings of this large study in children with biopsy-proven NAFLD are that: (1) simple noninvasive scores that have been validated to predict advanced fibrosis in adult patients with NAFLD are not accurate in children, (2) the newly developed PNFS is more accurate than APRI, FIB-4 index or NFS for predicting the presence of advanced fibrosis in children with fatty liver disease and may become a useful tool to determine patients that need more intensive evaluation and management.

NAFLD represents a disease spectrum that ranges from simple steatosis to non-alcoholic steatohepatitis (NASH), to advanced fibrosis and ultimately cirrhosis [24]. The presence and stage of fibrosis is considered an important factor in the prognosis of NAFLD and the prediction of the risk of developing cirrhosis [25]. Studies on natural history of pediatric NAFLD showed that fibrosis can progress to cirrhosis and its feared complications during childhood [3]. Therefore this brings the necessity of early diagnosis of advanced fibrosis in NAFLD to light as that can play an important role in preventing development of further complications [26]. The gold standard of diagnosis remains percutaneous liver biopsy which is not only a costly procedure but is invasive with high risk of complications and sampling error [27]. Several fibrosis scores have been developed and validated to predict advanced fibrosis in large studies on adults with NAFLD. In fact, the NFS was recommended as a clinically useful tool for identifying NAFLD patients with higher likelihood of having bridging fibrosis and/or cirrhosis by the recent practice guidelines endorsed by all the major gastroenterology and hepatology societies in the United States [4]. Furthermore, recent data demonstrated that these simple scoring systems can predict long-term outcomes of adult patients with NAFLD including liver-related complications and death with the best indicator being the NFS followed by APRI and FIB-4 scores [28]. However, none of these scores has been developed or validated in children with NAFLD. To date, only one fibrosis score has been developed by our group for children with NAFLD, namely the Pediatric NAFLD Fibrosis Index (PNFI) [29]. Due to the fact that the majority of children in the original PNFI cohort had only mild fibrosis (stage 1) and none had cirrhosis, we could not develop a predictor of advanced fibrosis. A recent study from South Korea that included 77 children with biopsy-proven NAFLD showed that the PNFI did not perform well for predicting significant fibrosis [6].

The ability of the new PNFS to separate children with advanced fibrosis may be explained by some of the variables included in the score. Low platelet count indicates more advanced liver disease and portal hypertension. ALT level is a surrogate for necroinflammatory activity and may server as a proxy for disease activity. Interestingly, alkaline phosphatase and GGT levels correlated with the presence of advanced fibrosis and further investigations are warranted to explain this phenomenon. If validated externally, PNFS may become a useful tool to rule in the presence of advanced fibrosis in children referred to large centers for management of fatty liver disease. By using a cutoff value that maximizes specificity, we propose that children with PNFS values above that cutoff should undergo a liver biopsy to confirm the presence of advanced fibrosis. This will minimize the number of children with no advanced fibrosis that will undergo liver biopsy. These children with mild disease do not need more aggressive monitoring for liver related morbidities during childhood and they should be encouraged to maintain a healthy lifestyle and lose weight if indicated. On the other hand, if the biopsy confirms the presence of advanced fibrosis in children with high PNFS, they should be monitored closely for the development of cirrhosis, complications of portal hypertension and potentially hepatocellular carcinoma.

We recognize that our study has several limitations, mainly related to selection bias and the fact that our study consisted of children followed at a tertiary hospital specialized in pediatric hepatology which may provide a skewed representation of the true prevalence of advanced fibrosis. Most of our children were white, making it difficult to determine the utility of PNFS among other ethnic groups. External cross-validation is needed before PNFS can be used to predict advanced fibrosis in children with NAFLD.

This study has several strengths including the large number of children with biopsy-proven NAFLD with adequate samples for interpretation by pathologist. Sampling issues associated with the quality of liver biopsies were excluded by not including any biopsy that was fragmented, <15 mm in length or had <6 portal tracts. The inclusion of a large number of children with advanced fibrosis (including two patients with cirrhosis) represents another unique strength of this study.

In conclusion, we have demonstrated clearly that noninvasive hepatic fibrosis scores developed in adults had poor performance in diagnosing advanced fibrosis in children with NAFLD. We have developed a new pediatric specific score called the PNFS, with improved performance characteristics. PNFS may help clinicians select children with NAFLD who are at high risk for advanced disease to undergo liver biopsy and more aggressive monitoring. The score must be validated in other populations before it can be recommended for this purpose.
